# Benign Breast Disease in Makkah, Saudi Arabia: A Retrospective Analytical Cross-Sectional Study

**DOI:** 10.7759/cureus.31174

**Published:** 2022-11-06

**Authors:** Mohammad I Almatrafi, Mutlaq A Almalki, Jumanah A Althagafi, Tala S AlSindi, Roaa M Masarit, Renad M Almatrafi

**Affiliations:** 1 College of Medicine, Umm Al-Qura University, Makkah, SAU; 2 Breast Surgery, Al-Noor Specialist Hospital, Makkah, SAU; 3 College of Medicine and Surgery, Umm Al-Qura University, Makkah, SAU

**Keywords:** fibrocystic changes, fibroadenoma, benign breast lesions, breast diseases, benign breast diseases

## Abstract

Introduction

The most frequent reason for visiting breast clinics is benign breast disease (BBD), which accounts for 90% of all breast-related presentations globally. It is widespread among women of childbearing age, peaking between the ages of 30 and 50. However, owing to the lack of studies on BBD compared with breast cancer in Saudi Arabia, this study aimed to assess the common patterns of BBD and factors associated with the frequency of fibroadenoma (FA) occurrence.

Methodology

A retrospective analytical study was carried out at Al-Noor Specialist Hospital, Makkah, Saudi Arabia, from May to August 2022. The sample was all patients who attended the breast and endocrine unit from January 2015 to December 2020.

Results

This study included 222 of 367 patients who had BBD. Of them, 42.3% were aged 31-45 years, with a mean age of 36.71 ± 12.48 years. The mean body mass index (BMI) was 26.45 ± 6.69 kg/m^2^, and the mean tumor size was 4.22 ± 4.9 mm.

Conclusion

Fibroadenoma among BBD types is the most common lesion in the studied population. This study established the baseline pattern of BBD in a specialized hospital in Makkah.

## Introduction

The most frequent reason for visiting breast clinics is benign breast disease (BBD), which accounts for 90% of all breast-related presentations globally [1,2]. BBD is widespread among women of childbearing age, peaking between the ages of 30 and 50 [3]. It is a range of histological entities that includes non-proliferative lesions, atypical hyperplasia, and proliferative lesions without atypia [4]. Although BBD is frequently benign, some cases do develop into cancer [1]. The patient’s medical history, clinical examination, and imaging modalities are all used to make the diagnosis [5].

Early menarche, regular short menstrual cycles, oral contraceptives, hormonal replacement therapy, nulliparity, pregnancy at an older age, and having a high postmenopausal body mass index (BMI) are risk factors linked to a higher risk of breast cancer, whereas having a high premenopausal BMI and nursing for a long time is linked to a lower risk [6-9]. Patients with BBD frequently exhibit mastalgia, a palpable tumor, and nipple discharge, necessitating biopsy in addition to targeted diagnostic imaging such as mammography, ultrasound, and magnetic resonance imaging (MRI) [5]. Up to 30% of women diagnosed with BBD will eventually need therapy [1].

Although some forms of BBD are linked to the development of later breast cancer, non-proliferative lesions with atypical hyperplasia are 3.9-13-fold more likely than proliferative lesions without atypia (1.3-1.9-fold greater risk) [10]. Between 70% and 79% of breast lumps in Uganda, Trinidad, and Nigeria are BBD, the majority of which are fibroadenoma (FA) and fibrocystic change (FCC) [11].

Similarly, the most common BBD in Saudi Arabia is FA, followed by FCC [12-14]. Owing to the lack of studies on BBD compared with breast cancer in Saudi Arabia, this study aimed to assess the common patterns of BBD and their demographic distribution at Al-Noor Specialist Hospital, Makkah, Saudi Arabia. It also aimed to assess the factors associated with the frequency of FA occurrence.

## Materials and methods

Study design, setting, and time

This retrospective cross-sectional study was carried out at Al-Noor Specialist Hospital, Makkah, Saudi Arabia, from May to August 2022.

Study population

The inclusion criteria were patients between 18 and 80 years who presented to the breast and endocrine unit of Al-Noor Specialist Hospital because of a breast complaint (mass, pain, swelling, and discharge) or for a screening mammogram from 2015 to 2020. The exclusion criteria were patients with malignancy, incomplete data, and pregnant or lactating women.

Sample size

The sample comprised all patients who attended the breast and endocrine unit from January 2015 to December 2020 and met the inclusion criteria.

Data collection

A predesigned checklist was prepared to collect patients’ information: age, gender, comorbidities (hypertension, diabetes mellitus (DM), chronic obstructive pulmonary disease, chronic kidney disease, and ischemic heart diseases), medications, type of surgery (lumpectomy, incisional biopsy, and excisional biopsy), type of biopsy (true cut, fine needle aspiration, and open biopsy), type of lesion found (FA, intraductal papilloma, benign phylloid tumor, lobular carcinoma in situ, fat necrosis, tubular adenoma, juvenile FA, lipoma, FCC, fibroadenomatoid hyperplasia, sclerosing adenosis, and others), modality of choice (mammogram, ultrasound, and MRI), and type of abnormality detected (calcification, lymph node (LN) enlargement, inflammatory changes, and mass).

Ethical considerations

Ethical approval for the study was obtained from the Institutional Review Board (IRB) of Al-Noor Specialist Hospital, Makkah, Saudi Arabia.

Data analysis

Data were analyzed using Statistical Package for the Social Sciences (SPSS) version 26 (IBM SPSS Statistics, Armonk, NY, USA). The chi-squared test (χ^2^) was applied to qualitative data expressed as numbers and percentages to examine the relationship between the variables. The Mann-Whitney U test was used to analyze non-parametric variables and quantitative data presented as mean and standard deviation (mean ± SD). A p-value of less than 0.05 was regarded as statistically significant.

## Results

This study included 222 patients who had BBD. Of them, 42.3% were aged 31-45 years, with a mean age of 36.71 ± 12.48 years. The mean BMI was 26.45 ± 6.69 kg/m^2^, and the mean tumor size was 4.22 ± 4.9 mm. Of the participants, 92.3% were women, and 82.4% had Saudi nationality. Only 8.6% had chronic diseases, of whom 26.3% had iron deficiency anemia. Almost half of the participants (50.9%) were taking medications (Table 1). The most frequent diagnosis was FA (55.4%) (Table 2).

**Table 1:** Distribution of participants according to their demographics, BMI, tumor size, chronic diseases, and medications (total number: 222) BMI, body mass index; mm, millimeter; CHD, congenital heart defects; DM, diabetes mellitus

Variable		Number (%)
Age (years)	<30	75 (33.8)
	31-45	94 (42.3)
	>45	53 (23.9)
Mean age (years)		36.71 ± 12.48
BMI		26.45 ± 6.69
Tumor size (mm)		4.22 ± 4.9
Gender	Female	205 (92.3)
	Male	17 (7.7)
Nationality	Non-Saudi	39 (17.6)
	Saudi	183 (82.4)
Chronic diseases	Yes	19 (8.6)
	No	203 (91.4)
Chronic diseases in the studied population (number: 19)	Anxiety, depressive episodes	1 (5.2)
	CHD	1 (5.2)
	Chronic adenotonsillitis	2 (10.5)
	Asthma	1 (5.2)
	Infertility	1 (5.2)
	DM	3 (15.7)
	Hypothyroidism	3 (15.7)
	Iron deficiency anemia	5 (26.3)
	Renal failure	1 (5.2)
	Scoliosis	1 (5.2)
Medication	Yes	113 (50.9)
	No	109 (49.1)

**Table 2:** Distribution of patients according to their diagnosis (total number: 222) *The most common diagnosis

Diagnosis	Number (%)
Abscess	18 (8.1)
Benign phyllodes tumor	9 (4.1)
Fibroadenoma*	123 (55.4)
Fibrocystic changes	34 (15.3)
Gynecomastia	12 (5.4)
Hyperplasia	2 (0.9)
Keratinous cyst	2 (0.9)
Lipoma	7 (3.2)
Mastitis	8 (3.6)
Papilloma	1 (0.5)
Schwannoma	2 (0.9)
Sclerosing adenosis	1 (0.5)
Sebaceous cyst	1 (0.5)
Tubular adenoma	2 (0.9)

For 37.8% of the patients, the tumor was either on the left or on the right side equally. One-third (33.3%) had bilateral benign-looking LN. Of the documented Breast Imaging Reporting and Data System (BI-RADS) scores, 12.1% had a score of 3. Over half (55%) of the patients had an excisional biopsy. Of the patients, 33.3%, 82.4%, and 9.9% underwent mammograms, ultrasounds, and MRI, respectively (Table 3).

**Table 3:** Distribution of patients according to clinical data and investigations (total number: 222) BI-RADS, Breast Imaging Reporting and Data System; US, ultrasound; MRI, magnetic resonance imaging *The most common diagnostic imaging technique used

Variable		Number (%)
Tumor side	Left	84 (37.8)
	Right	84 (37.8)
	Both	48 (21.6)
	Not available	6 (2.7)
Lymph node	Not available	129 (58.1)
	Bilateral benign-looking	74 (33.3)
	Left axillary	5 (2.3)
	Right axillary	14 (6.3)
BI-RADS score	1	4 (1.8)
	2	8 (3.6)
	3	27 (12.1)
	4	24 (10.8)
	Not available	159 (71.7)
Biopsy	Not available	43 (19.4)
	Excision	123 (55)
	Fluid needle aspiration	3 (1.4)
	Incision and drainage	5 (2.3)
	Lumpectomy	11 (5)
	US-guided true-cut biopsy	37 (17)
Mammogram	Not done	148 (66.7)
	Done	74 (33.3)
US*	Not done	39 (17.6)
	Done	183 (82.4)
MRI	Not done	200 (90.1)
	Done	22 (9.9)

**Figure 1:**
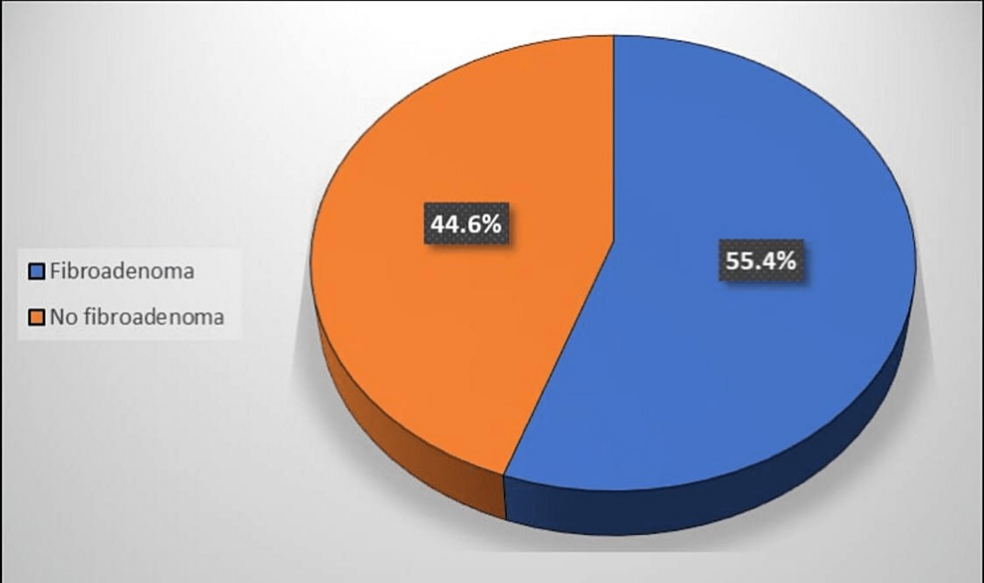
Percentage of patients according to FA (total number: 222) FA, fibroadenoma

**Figure 2:**
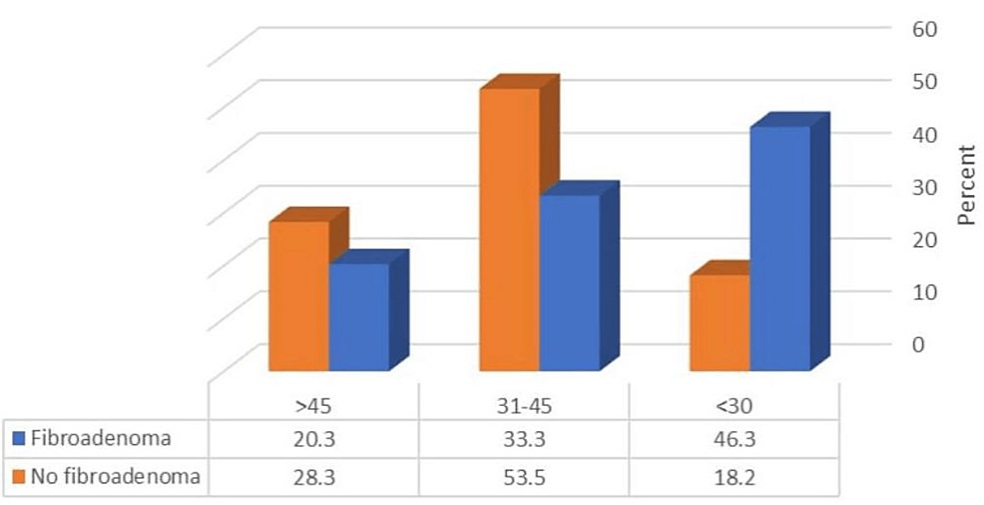
Relationship between FA and patients’ age (total number: 222) FA, fibroadenoma χ^2^: 19.61, p-value ≤ 0.001

Figure 1 illustrates that 55.4% of the studied patients were diagnosed with FA. Additionally, FA was significantly higher among patients aged under 30 years (p ≤ 0.05) (Figure 2). Table 4 shows a significantly higher rate of FA among patients in their 30s (p ≤ 0.05).

**Table 4:** Relationship between FA and patients’ demographics, BMI, tumor size, chronic diseases, and medications (total number: 222) FA, fibroadenoma; SD, standard deviation; BMI, body mass index; mm, millimeter *Mann-Whitney U test

Variable	Fibroadenoma		χ^2^	p-value
	Yes (number (%))	No (number (%))		
Age (years) (mean ± SD)	33.55 ± 12.28	40.64 ± 11.65	1*	<0.001
BMI	26.08 ± 7.03	26.97 ± 6.2	1.23*	0.218
Tumor size (mm)	4.48 ± 5.68	3.77 ± 3.01	0.53*	0.596
Gender			14.19	<0.001
Female	121 (98.4)	84 (84.8)		
Male	2 (1.6)	15 (15.2)		
Nationality			0.85	0.355
Non-Saudi	19 (15.4)	20 (20.2)		
Saudi	104 (84.6)	79 (79.8)		
Chronic diseases			0.05	0.819
Yes	11 (8.9)	8 (8.1)		
No	112 (91.1)	91 (91.9)		
Medication			0.83	0.36
Yes	66 (53.7)	47 (47.5)		
No	57 (46.3)	52 (52.5)		

## Discussion

The present study was carried out to investigate the patterns of BBD and its associated risk factors in Makkah. Our findings showed that among the 222 participants, FA was the most common BBD, followed by FCC, abscesses, and gynecomastia. Furthermore, the prevalence of FA was higher in young women than in other subjects.

Compared with previous studies, FA remains the most frequently reported type of BBD (55.4%). Similar observations were reported by Albasri [12], Chiedozi et al. [15], and Jamal [13], at 44.3%, 27.5%, and 47%, respectively. These findings align with most of the literature, including the studies of Malik et al. [16] and Aslam et al. [17].

In this study, FA was highly prevalent in the third decade, with a mean age of 33.55 years, similar to the research of Razik et al. [18] and higher than the study of Albasri [12]. However, other variables, such as residence in Saudi Arabia and chronic diseases, were not significantly associated with FA, which disagrees with the study of Razik et al. [18].

The similarity of our result with those mentioned above is consistent with national studies regardless of the level of care. Our research was carried out in a specialized center, whereas the study of Albasri [12] was in a tertiary hospital, the study of Razik et al. [18] was a multicentric study, and Jamal [13] was in a teaching hospital.

In our study, FCC was the second most commonly reported lesion, accounting for 15.3% of all BBD types. This finding is in line with the findings of Albasri [12], Alamri et al. [19], and Amr et al. [20]. They found an FCC frequency of 23.4%, 18.8%, and 21.1%, respectively.

In our cohort, abscesses were the third most commonly reported BBD type, accounting for 8.1%, which is comparable to the study of Alamri et al. (5.9%) [19] and higher than the study of Ahmad Mohammed (0.8%) [21]. In the present study, benign phylloid tumor was found in 4.1% of the study population, while Aslam et al. [17] and Masciadri and Ferranti [22] reported a lower rate. Other BBD types, such as gynecomastia, epithelial hyperplasia, lipoma, and mastitis, were found to be low in our cohort; similar rates were also reported in the studies of Albasri [12] and Razik et al. [18].

Limitations

The study has some limitations, notably its retrospective design and sample size affected by poor recording systems, which limited data collection to a five-year period. The lower rate of benign breast disease in our study compared with the Makkah population may result from many variables such as the distribution of patients across city hospitals. However, other reasons could explain our findings such as a lack of awareness of such conditions, hesitancy to seek medical care due to social difficulties, and ignorance of these issues.

## Conclusions

In conclusion, fibroadenoma is the most common lesion of benign breast disease in the studied population. This study established the baseline pattern of benign breast disease in a specialized hospital in Makkah, which provides valuable information to local physicians.
